# All-photonic drying and sintering process *via* flash white light combined with deep-UV and near-infrared irradiation for highly conductive copper nano-ink

**DOI:** 10.1038/srep19696

**Published:** 2016-01-25

**Authors:** Hyun-Jun Hwang, Kyung-Hwan Oh, Hak-Sung Kim

**Affiliations:** 1 Department of Mechanical Engineering, Hanyang University, Haengdang-dong, Seongdong-gu, Seoul 133-791, South Korea; 2 Institute of Nano Science and Technology, Hanyang University, Seoul, 133-791, Korea

## Abstract

We developed an ultra-high speed photonic sintering method involving flash white light (FWL) combined with near infrared (NIR) and deep UV light irradiation to produce highly conductive copper nano-ink film. Flash white light irradiation energy and the power of NIR/deep UV were optimized to obtain high conductivity Cu films. Several microscopic and spectroscopic characterization techniques such as scanning electron microscopy (SEM), a x-ray diffraction (XRD), and Fourier-transform infrared (FT-IR) spectroscopy were employed to characterize the Cu nano-films. Optimally sintered Cu nano-ink films produced using a deep UV-assisted flash white light sintering technique had the lowest resistivity (7.62 μΩ·cm), which was only 4.5-fold higher than that of bulk Cu film (1.68 μΩ•cm).

In the past decade, interest in printed electronics had increased^1^,^2^,^3^,^4^,^5^. Printed electronics refers to the application of printing technologies to fabricate electronic circuits and devices such as radio frequency identification (RFID) tags^6^, wearable electronics^7^, and large-area displays. In particular, realization of such devices on flexible (bendable plastic or paper) substrates is becoming very important for commercialization of low cost printed electronics devices^8^,^9^,^10^. Conventionally, electronic devices are fabricated by photolithography and vacuum deposition processes. However, these methods are multi-stage (nine steps), require expensive equipment, and involve the use of environmentally undesirable chemicals, resulting in the generation of large amounts of waste. Printed electronics are faster, cheaper, and simpler to synthesize (two-step process) than those synthesized using conventional production methods. Furthermore, printed electronics can be combined with inkjet printing or the roll-to-roll (R2R) technique, which makes the process economically feasible^11^,^12^,^13^.

Metallic nano-inks made with nanoparticles have been widely employed because of their low melting point and unique properties^14^,^15^,^16^,^17^,^18^. Recently, copper nano-inks have been developed as a low-cost alternative to silver or gold nano-inks for printed electronics^19^. However, most copper nanoparticles are covered with an oxide shell, and cannot be sintered by thermal sintering under ambient conditions. Several approaches such as the laser process^20^ and plasma process^21^ have been developed to sinter copper nanoparticles without oxide shells. However, these approaches have limitations in mass production because of their low throughput, high complexity, and considerable environmental obstacles (e.g. requirement for vacuum conditions and chamber). Furthermore, a laser sintering process can cover only a small sintering area; a sophisticated 3D-gantry system is required to cover a large area. Therefore, a new low temperature and rapid large area sintering technique is required to realize mass production of flexible electronic devices on polymer or paper substrates.

To satisfy this demand, we previously developed a flash white light (FWL) sintering method combined with poly (*N*-vinylpyrrolidone) (PVP) functionalization of copper nanoparticles^22^,^23^,^24^,^25^. Flash white light sintering can instantly reduce the copper oxide shell and sinter copper nanoparticles at room temperature under ambient conditions in a few milliseconds without damaging the substrate. In addition, it is possible to sinter a large area of Cu nanofilm using flashes from a xenon lamp. However, an in-depth study of photonic sintering characteristics using multiple light sources for PVP functionalized Cu nanoparticle ink has not yet been conducted.

In this study, we investigated the effects of drying conditions on the flash white light sintering of copper nano-ink using near infrared (NIR) irradiation at different temperatures (80 °C and 120 °C). Furthermore, NIR- or deep UV-assisted flash white light sintering of Cu nano-ink was demonstrated by irradiating the films with NIR and deep UV during the flash white light sintering process for a few milliseconds (Fig. 1a). To verify the mechanisms of photonic sintering of Cu nano-ink, temperature and electrical resistance changes during the deep UV-assisted flash light sintering process were monitored using a Wheatstone bridge electrical circuit and a high-rate data acquisition system. The effects of flash white light irradiation energy and the power of NIR/deep UV irradiation on the sintering of copper nano-ink were studied using several microscopic and spectroscopic characterization techniques such as SEM, XRD, and FT-IR.

## Results

### Drying temperature effect on Flash white light sintering of Cu nano-ink

In our previous studies, we found that Cu nano-ink film can be sintered by flash white light with an irradiation energy higher than 7.5 J/cm^2^, but was damaged when the irradiation energy was higher than 12.5 J/cm^2^ 24,25. Therefore, to sinter Cu nano-ink films, we varied the irradiation energy of the flash white light from a xenon lamp from 7.5 J/cm^2^ to 12.5 J/cm^2^; one pulse of flash white light with a duration of 10 ms was used. As shown in Fig. 2a, the resistivity of the Cu nano-ink films dried by NIR irradiation at 80 °C (N80) and 120 °C (N120) decreased as the irradiation energy increased up to 12.5 J/cm^2^. These results are consistent with those reported in our previous studies^24^,^25^. We also found that a higher drying temperature (120 °C) decreased the resistivity of the flash light-sintered Cu films relative to films produced at a lower drying temperature (80 °C). However, at an irradiation energy of 12.5 J/cm^2^, there was no significant difference between the resistivities of the Cu films dried at 80 °C and 120 °C (Fig. 2a).

SEM surface micrographs of the Cu films before- and after- sintering by flash white light and deep UV irradiation are shown in Fig. 3. In unsintered Cu nano-ink films, the Cu nanoparticles were surrounded by PVP binder, which resulted in blurry SEM images (Fig. 3a,d). With flash white light irradiation, the PVP binder was decomposed and evaporated, allowing the copper nanoparticles to be observed more clearly (Fig. 3b,e). Simultaneously, the copper nanoparticles were sintered by flash white light. The average grain size of Cu nanoparticles increased and the neck-like junctions among Cu nanoparticles grew larger with flash white light irradiation (Fig. 3b,e), resulting in an increase in conductivity.

Meanwhile, the adsorption bands of PVP (C-N; 1294 cm^−1^ ν, CH_3_; 1423 cm^−1^ν, and CH_2_; 1461 cm^−1^ν) and the stretching carbonyl groups (C=O; 1652 cm^−1^ν) attached to the end of the PVP chain in the FT-IR spectra (Fig. 2b) were more pronounced in the lower drying temperature film (N80) before flash white light sintering than the higher drying temperature film (N120). This means that a large amount of polymer binder remained in the Cu films dried at the lower temperature due to low heat energy supply at a drying temperature of 80 °C (N80). A large amount of the residual polymer binder disturbed the sintering of Cu nanoparticles and caused more inter-granular pores after flash white light sintering (Fig. 3b), while pores were rarely observed in the Cu films dried at 120 °C (N120) (Fig. 3e). For these reasons, a lower drying temperature resulted in a sintered Cu film with higher resistivity than that of the film obtained at a higher drying temperature (N120) (Fig. 2a). However, as mentioned above, at an irradiation energy of 12.5 J/cm^2^, the flash white light evaporated residual organic binder and sintered the copper nanoparticles sufficiently to obtain high conductivity, even when dried at a low temperature (Fig. 2a).

### NIR-assisted Flash white light sintering of Cu nano-ink

NIR ranging in power from 1 W/cm^2^ to 4 W/cm^2^ at a distance of 15 cm was also applied during the flash white light sintering of Cu nano-ink. As shown in Fig. 4a, the resistivity of the sintered Cu film decreased as the NIR irradiation power increased up to 3 W/cm^2^. However, at 4 W/cm^2^ of NIR irradiation, the resistivity of the Cu film increased again. To verify the effect of NIR irradiation on flash white light sintering and determine the underlying mechanism, temperature changes during the NIR-assisted flash white light sintering process were monitored and recorded (Fig. S1). As shown in Fig. 4b, the temperature of the Cu nano-ink film increased instantly with flash white light irradiation. The maximum temperature of the copper nanoparticle film was 250 °C (see black line in Fig. 4b), allowing complete vaporization of the PVP and melting of the Cu nanoparticles. Diameters of the copper nanoparticles used in this work varied from 20 nm to 60 nm, suggesting an average melting temperature of about 215 °C^26^. After flash light irradiation ended, the melted liquid Cu nanoparticles started to cool and transform to solid phase, dissipating heat from the copper to the surrounding air^27^.

When flash white light irradiation was combined with NIR irradiation, the temperature of the Cu nano-film increased up to about 300 °C (see red line in Fig. 4b), which was higher than that of the flash white light irradiation-only case (black line in Fig. 4b). According to the evolution of microstructures, the sintering process can be divided into four stages: activation and refinement of the Cu nanoparticles, formation and growth of the sintering neck, rapid densification, and plastic deformation densification. Temperature is one of the dominant thermodynamic variables related to sintering conditions. Sintering at a higher temperature enhances the densification rate relative to the grain growth rate^28^,^29^. Thus, when a high sintering temperature was applied to the Cu nanoparticles, high-quality bulk copper was obtained. For this reason, NIR-assisted flash white light irradiation resulted in Cu films with lower resistivity and larger grain boundaries than those obtained with flash white light irradiation only (see inset figures in Fig. 4b). However, when the power of NIR was higher than 4 W/cm^2^, the Cu films were damaged and the resistivity of the Cu films increased (Fig. 4a).

### Deep UV-assisted Flash white light sintering of Cu nano-ink

To further enhance the efficiency of the photonic sintering of Cu nano-ink, deep UV irradiation was also applied with flash white light sintering. The power of deep UV irradiation was varied from 30 mW/cm^2^ to 90 mW/cm^2^ while films were irradiated with one 10-ms pulse of 12.5 J/cm^2^ flash white light. As shown in Fig. 5, application of deep UV irradiation during the flash white light sintering process resulted in a considerable decrease in resistivity of the sintered Cu films. We also observed that Cu nanoparticles irradiated by the flash white light combined with deep UV agglomerated more densely than those irradiated by flash white light only, as shown in the SEM images (Fig. 5a,b). It is noteworthy that deep UV irradiation at a power of only 10–100 mW, which is about 4–5 orders lower than that of the flash white light (1.25 kW) in this study, decreased the resistivity of the Cu films considerably. This is because poly (*N*-vinylpyrrolidone) (PVP) is decomposed very effectively by deep UV irradiation because of its UV (198 nm) absorption characteristics^30^. Previously, Ryu *et al.* proposed a mechanism for flash white light sintering of Cu nanoparticles covered with a PVP-functionalized oxide shell^23^. They proposed that when copper nanoparticles are exposed to flash white light, the high temperature of the nanoparticles results in superheating of their surfaces and instantaneous generation of energetic copper ions^31^,^32^. Simultaneously, flash white light irradiation converts the PVP chains to primary alcohols, secondary alcohols, carboxyl acid, or acetic acid through photo-degradation phenomena involving OH radical attack, as shown in Fig. 6^30^,^33^. Both alcohol and acid reduction mechanisms are generic routes for synthesis by metal oxide reduction that have been used for decades^34^,^35^,^36^. The intermediate alcohols and acid resulting from photo-thermal degradation of PVP might reduce the copper oxide shell of the superheated copper nanoparticles. For these reasons, opening of the pendant lactam ring by UV irradiation may be the most important feature in the photodecomposition of PVP structures^30^,^33^, resulting in the formation of intermediate alcohols and acids and followed by reduction of the copper oxide shell as the ultimate step. Thus, we hypothesize that deep UV irradiation during flash white light irradiation enhanced the reduction and sintering of Cu nano-ink because the PVP decomposed more effectively under UV irradiation^30^. However, the resistivity of the sintered Cu films increased again as the power of deep UV irradiation increased. This is because excessive photodecomposition of PVP at a UV irradiation power of more than 60 mW/cm^2^ damaged the Cu nano-films (Fig. 5c,d).

To study the deep UV-assisted flash white light sintering of Cu nanoparticles in greater detail, we monitored the sheet resistance changes of Cu films in real time during the sintering process using a Wheatstone bridge electrical circuit and oscilloscope (flash white light: 12.5 J/cm^2^ irradiation energy, 1 pulse of 10 ms pulse duration, 30 mW/cm^2^ of deep UV power) (Fig. S1). As shown in Fig. 7a, the *in situ* monitoring results revealed that sheet resistance decreased to 1.7 Ω/sq immediately after flash white light irradiation. In this interval, the Cu oxide shell surrounding the Cu nanoparticles was likely reduced and sintered by flash light irradiation followed by PVP decomposition^23^,^24^. However, the sheet resistance of the flash light sintered Cu nano-ink film increased slightly at about 50 milliseconds after irradiation. This may be because the reduced and sintered Cu nanoparticles were oxidized again by reaction with oxygen in air. As shown in the SEM image in Fig. 5a, Cu film sintered by only flash white light still had a few pores, enabling copper nanoparticles to be oxidized easily. Thus, the large surface area of Cu film with nanoparticles caused the oxidation of copper after flash white light sintering. XRD pattern shown in Fig. 7b demonstrated that copper oxide (Cu_2_O, copper (I) oxide) peaks (36.4° and 38°) were still observed even after flash white light irradiation, although those peaks decreased in intensity. Deep UV-assisted flash white light sintering led to a larger decline in the sheet resistance of Cu film than flash white light irradiation only. The increase in sheet resistance due to oxidation was not observed, even after several seconds. This may be because of the smaller surface area of the sintered Cu film, which had a smooth surface and few pores (Fig. 5b). Copper oxide peaks were rarely observed and the intensity of pure Cu phase peaks (43.2°, 50.4° and 74.1°) increased considerably after irradiation with flash white light and deep UV (Fig. 7b). These findings are consistent with the SEM images (Fig. 5b) and *in situ* monitoring results (red line in Fig. 7a).

Figure 7d shows the effect of different NIR drying temperatures (N80 and N120) on the Cu nano-ink films. In this case, the parameters used in the deep UV-assisted flash white light sintering process were fixed at optimized values (30 mW/cm^2^ of deep UV and flash light sintering with one pulse of 10 ms duration). The deep UV-assisted flash white light sintered Cu nano-ink films (FWL+UV) showed lower resistivity than the flash white light sintered films (FWL). It is noteworthy that the resistivity of the Cu nano-ink film increased after sintering in the case of films dried at the higher NIR drying temperature of 120 °C (N120). This might be because too much PVP binder evaporated during drying at the higher NIR temperature (see FT-IR results in Fig. 2b). The smaller amount of PVP that remained in film dried at the NIR temperature of 120 °C could not entirely reduce the Cu oxide shells, as shown in the SEM image (Fig. 3d). Thus, the Cu film was damaged and pore size increased after sintering (Fig. 3f). These results correspond to those obtained from XRD analysis; the intensity of the Cu oxide shell peak (36.4°) increased and that of the pure Cu peak (43.2°) decreased in films generated by deep UV irradiation during the flash white light sintering process (Fig. 7c). Meanwhile, the lowest resistivity Cu film was obtained after flash white light sintering combined with deep UV irradiation and NIR drying at 80 °C (Fig. 8). The XRD pattern of the optimally dried (NIR drying at 80 °C) and sintered (flash light: 12.5 J/cm^2^, 1 pulse, 10 ms, deep UV power: 30 mW/cm^2^) Cu nano-ink was characterized by a pure Cu peak (43.2°) without any Cu oxide (36.4°) peak due to complete reduction of the Cu oxide shells and crystallization of the pure Cu phase. Furthermore, as shown in Fig. 8d, the optimized conductive Cu nano-film had a smooth surface similar to that of bulk copper phase, and pores were rarely observed. Finally, highly conductive Cu films with a resistivity of 7.62 μΩ•cm were successfully produced by NIR drying at 80 °C (N80) and deep UV-assisted flash white light sintering; this resistivity is only 4.5-fold higher than that of bulk copper (1.68 μΩ•cm).

When films were irradiated with NIR and deep UV in combination during the flash white light sintering process, the resistivity of the Cu film increased considerably (Fig. 8). This might be because excessive irradiation energy from deep UV and NIR damaged the Cu films, resulting in films with a relatively large porosity, as shown in the SEM image in Fig. 8e.

## Discussion

In order to reduce and sinter Cu nanoparticles with oxide shell efficiently, the effect of NIR drying temperature on flash white light sintering of the PVP functionalized Cu nano-ink was investigated. We found that a higher drying temperature (120 °C) could decrease the resistivity of the flash light sintered Cu films relative to films produced at a lower drying temperature (80 °C). However, at an irradiation energy of 12.5 J/cm^2^, the flash white light could evaporate residual organic binder and sintered the copper nanoparticles sufficiently to obtain high conductivity, even when dried at a low temperature. Thus, we concluded that there is no significant difference between the resistivities of Cu films dried at 80 °C and 120 °C using a NIR drying process if the Cu nano-ink film is fully sintered.

The effects of NIR irradiation during the flash white light sintering of Cu nano-ink were also studied. The resistivity of the Cu film sintered by NIR-assisted flash white light sintering process was decreased, as the NIR irradiation power increased up to 3 W/cm^2^. This was because, when flash white light irradiation was combined with NIR irradiation, the maximum temperature of the Cu nano-film during the sintering process was higher than that of the flash white light irradiation-only case. However, when the power of NIR was higher than 4 W/cm^2^, the Cu films were damaged and the resistivity of the Cu films increased, due to excessively high temperature. These results demonstrated that NIR irradiation during the flash white light sintering process enhanced the sintering characteristics of copper nano-ink. Note that although NIR alone has been used to sinter metal nanoparticles such as gold and silver by irradiating these nanoparticles for several minutes^37^,^38^, the sintering process is time-consuming for application to a roll-to-roll system.

Meanwhile, deep UV irradiation during flash white light sintering could further enhance the reduction and sintering of the Cu oxide shell, because the PVP coating layer decomposed more effectively under deep UV irradiation. Photonic drying (NIR 80 °C) and deep UV-assisted flash white light sintering process developed in this study (flash white light: 12.5 J/cm^2^, 1 pulse, 10 ms, deep UV power: 30 mW/cm^2^) yielded highly conductive Cu films at room temperature under ambient conditions in a few milliseconds. These films had a resistivity of 7.62 μΩ•cm, which is only 4.5-fold higher than that of bulk copper (1.68 μΩ•cm).

The photonic drying and sintering technology described for Cu nano-ink in this study is more effective than conventional drying and sintering method^20^,^21^,^39^,^40^ because of fast, efficient sintering and drying. Conductivity comparable to that reported in the literature^11^,^25^,^41^,^42^,^43^,^44^ was obtained by applying a flash white light sintering method combined with NIR and deep UV irradiation at a low drying temperature and short treatment time (Fig. 8f). Flash white light sintering reduced the oxide shell of copper nanoparticles at room temperature under ambient conditions in a few milliseconds. Furthermore, deep UV irradiation during flash white light sintering enhanced the sintering characteristics of PVP-functionalized Cu nano-ink, even though the irradiation power of deep UV is 4~5 orders lower than that of flash white light; this could reduce energy consumption during the sintering process. The novel photonic sintering technique for Cu nano-ink described here is a viable approach to realize room temperature *in situ* sintering of printed electronics.

## Methods

### Material preparation & fabrication of Cu nano-ink

To fabricate Cu nano-inks, we used commercially-available Cu nanoparticles with oxide shells (20–50 nm in diameter, oxide thickness >2 nm; QSI-Nano). Copper nanoparticles (11.4 g) were dispersed in a mixed solvent of diethylene glycol (DEG, 99%; Samchun Chemical) (4.5 g) and poly (*N*-vinylpyrrolidone) (PVP, MW 55,000; Sigma Aldrich) (0.9 g). To disperse copper nanoparticles, a mechanical stirrer and ultra-sonicator were used simultaneously for 3 hours. A three-roll mill was then used for 30 min to disperse copper nanoparticles in the highly viscous solvent. Cu nano-ink had a high viscosity (800~1000 centistokes) for printing on the PI substrate (solid content: 60~65%).

### Printing & drying of Cu nano-ink

The prepared Cu nano-ink was printed on polyimide (PI) substrate using the doctor blade method. Printed Cu nano-ink films on PI substrate were dried by near infrared lamp (NIR, wavelength rage: 800~1500 nm, 500W; Adphos L40). Samples were heated under the NIR lamp. The temperature of the Cu nano-ink film during the NIR drying process was recorded using a K-type thermocouple and samples were heated to different drying temperatures (80 °C and 120 °C) by controlling the NIR power.

### Photonic sintering of Cu nano-ink using flash white light combined with NIR and deep UV

**Figure 1:**
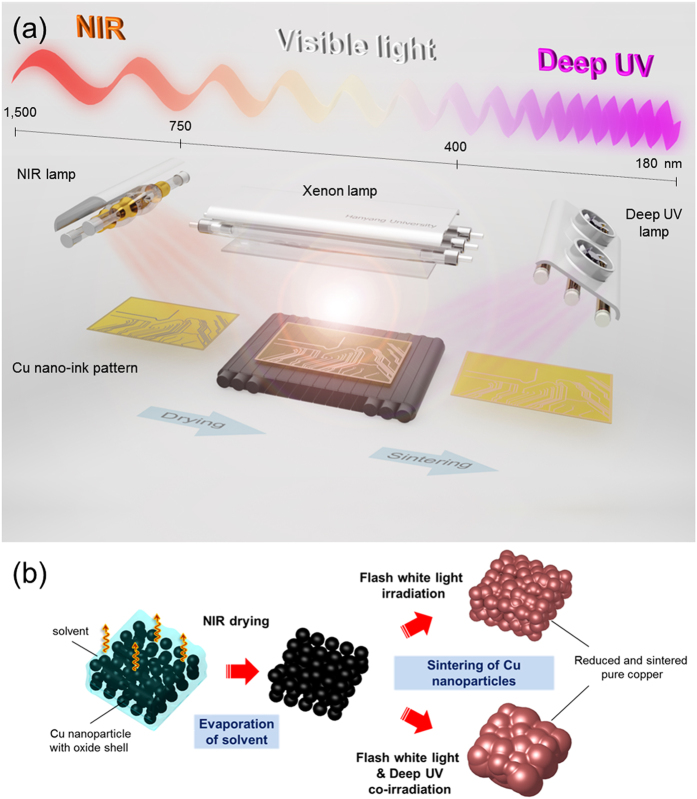
Schematics of the photonic drying and sintering process of Cu nano-ink, using NIR, flash white light, and deep UV. (**a**) Schematic view of all-photonic drying and sintering process of Cu nano-ink. (**b**) The morphology change of Cu nanoparticles in nano-ink during the photonic drying and sintering process.

**Figure 2:**
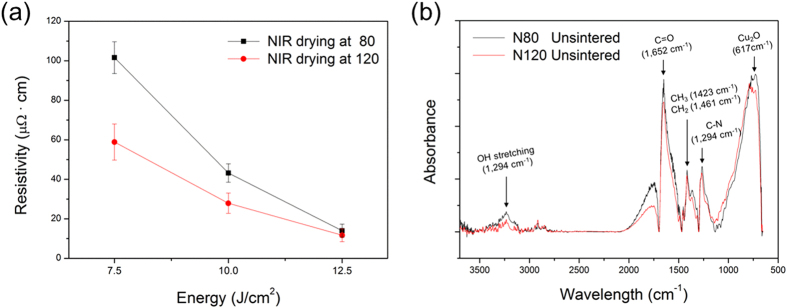
The effect of NIR drying temperature on the flash white light sintering of Cu nano-ink. (**a**) The resistivity of the flash light sintered Cu nano-ink film as increasing the irradiation energy from 7.5 J/cm^2^ to 12.5 J/cm^2^. (**b**) FTIR spectra of the Cu nano-ink film dried by NIR drying with different drying temperature (80 °C and 120 °C).

**Figure 3:**
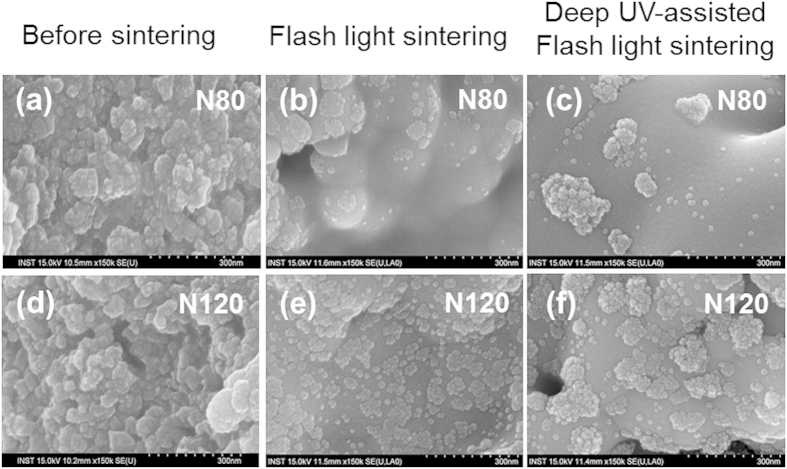
The SEM images of the Cu films before- and after- sintering by flash white light (Irradiation energy: 12.5 J/cm^2^, pulse duration: 10 ms, pulse number: 1) and deep UV (Irradiation power: 30 mW/cm^2^) irradiation.

**Figure 4:**
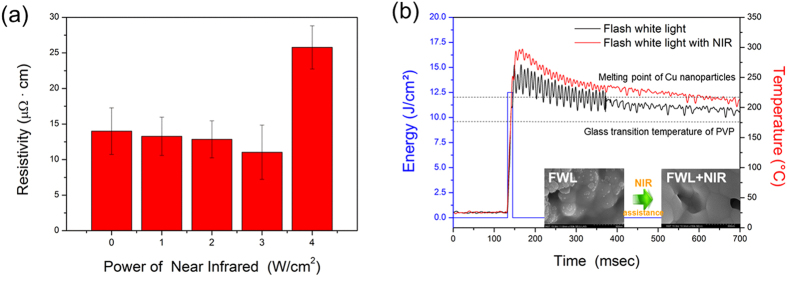
NIR-assisted flash white light sintering of Cu nano-ink. (**a**) The effect of NIR irradiation power on the sintering of Cu films during the flash light sintering process (Irradiation energy of flash white light: 12.5 J/cm^2^). **(b)** *In situ* monitoring of temperature change of Cu films during the photonic sintering process; (inset: SEM images of the Cu nano-ink sintered by flash white light only (irradiation energy : 12.5 J/cm^2^, pulse duration : 10 ms, pulse number : 1) and flash white light combined with NIR irradiation (3 W/cm^2^), respectively).

**Figure 5:**
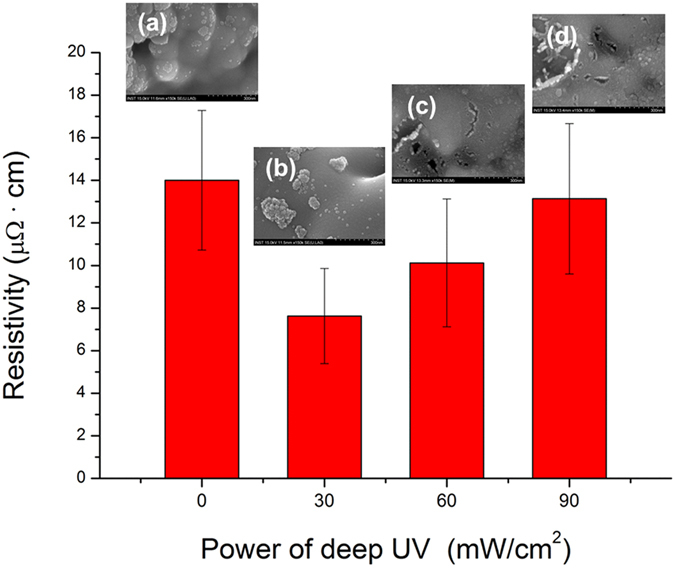
The effect of deep UV irradiation power on the sintering of Cu films during the flash light sintering process (irradiation energy: 12.5 J/cm^2^, pulse duration: 10 ms, pulse number: 1).

**Figure 6:**
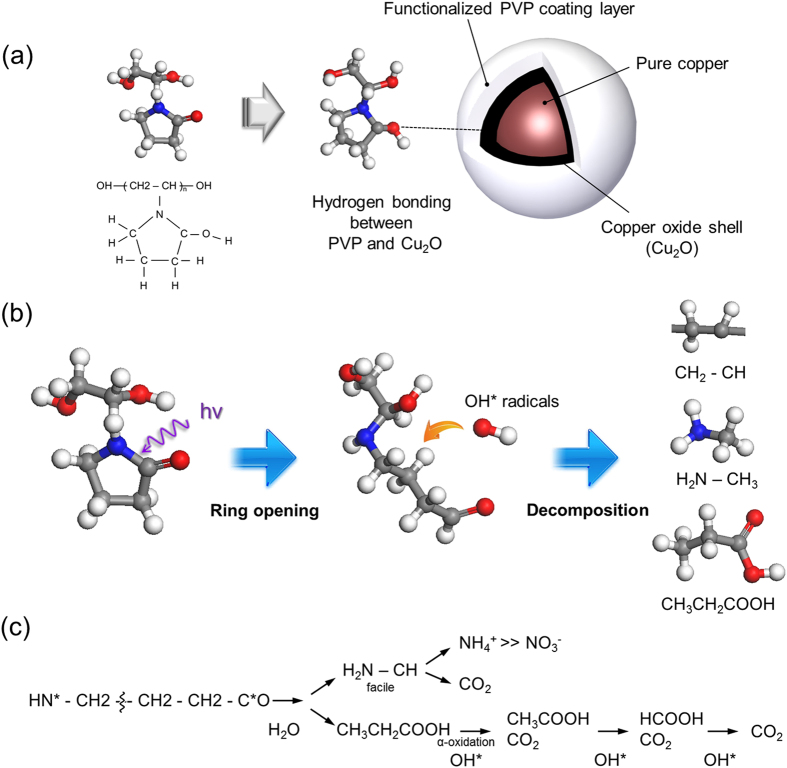
The schematics of the photo-degradation of PVP surrounding Cu nanoparticles and copper oxide shell, resulting in alcohol or acid.

**Figure 7:**
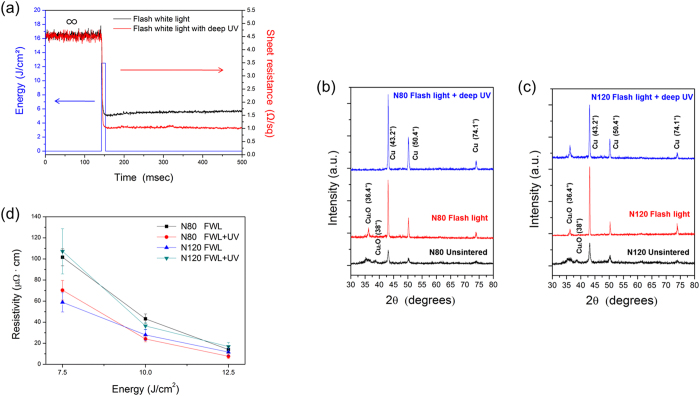
Deep UV-assisted flash white light sintering of Cu nano-ink (flash white light irradiation energy: 12.5 J/cm2, pulse duration: 10 ms, pulse number: 1, deep UV irradiation: 30 mW/cm2). (**a**) *In situ* monitoring of sheet resistance change of Cu films during the photonic sintering process. **(b)** XRD patterns of the unsintered and sintered Cu nano-ink film (N80) using flash white light and deep UV irradiation. **(c)** XRD patterns of the unsintered and sintered Cu nano-ink film (N120) using flash white light and deep UV irradiation. **(d)** The resistivity of the Cu nano-ink film sintered by flash white light irradiation combined with deep UV irradiation (30 mW/cm^2^).

**Figure 8:**
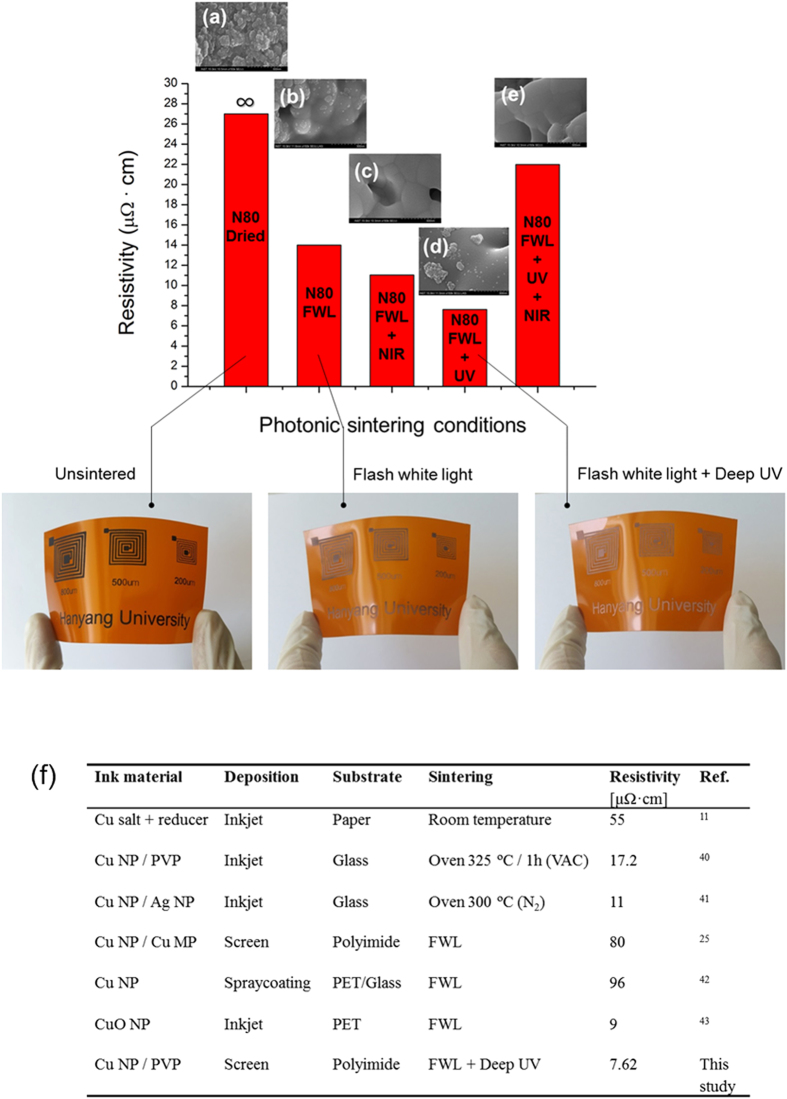
Comparison of the resistivity of the Cu films sintered by different sintering methods (Flash white light: 12.5 J/cm^2^ irradiation energy, 1 pulse number, 10 ms pulse duration, the power of deep UV: 30 mW/cm^2^, and the power of NIR: 3 W/cm^2^).

Printed Cu nano-ink films were sintered by flash white light combined with NIR and deep UV irradiation at room temperature under ambient conditions (Fig. 1a). The flash white light sintering system consisted of a xenon flash lamp (PerkinElmer Co.), a power supply, capacitors, a simmer triggering controller, a pulse controller, and a water cooling system. White light from a xenon flash lamp has a broad wavelength range from 380 nm to 950 nm. In addition, a commercial deep UV system (100 mW, LUMATEC SUV-DC) with a wavelength range from 180 nm to 280 nm and NIR system (500 W; Adphos L40) with a wavelength range of 800 nm to 1500 nm were used in this study. To optimize photonic sintering conditions, the irradiation energy of the flash white light and the power of NIR/deep UV were varied.

### Characterization

The microstructures and surfaces of the sintered Cu nano-ink films were examined by scanning electron microscopy (SEM, S4800 Hitachi). To investigate the oxidation or reduction of the oxide-covered copper nanoparticles, crystal phase analysis was performed by x-ray diffraction (XRD, D/MAX RINT 2000, CuK radiation). Fourier transform infrared (FT-IR) spectroscopy was used to determine whether the organic binder remained in the Cu nano-ink films or not. The sheet resistance of the Cu films was measured using a four-point probe method, followed by measuring the thickness of films through Alpha step (KLA Tencor AS500) to calculate resistivity (Fig. S5) .

## Additional Information

**How to cite this article**: Hwang, H.-J. *et al.* All-photonic drying and sintering process *via* flash white light combined with deep-UV and near-infrared irradiation for highly conductive copper nano-ink. *Sci. Rep.* **6**, 19696; doi: 10.1038/srep19696 (2016).

## Supplementary Material

Supplementary Information
