# Supplementary material for: All-photonic drying and sintering process via flash white light combined with deep-UV and near-infrared irradiation for highly conductive copper nano-ink
Source: Sci Rep. 2016 Jan 25;6:19696. doi: 10.1038/srep19696 (PMC4726351; doi:10.1038/srep19696)
Supplement: Supplementary Information [file srep19696-s1.pdf]

Supplementary Information

All-photonic drying and sintering process *via* flash white light combined with deep-UV and near-infrared irradiation for highly conductive copper nano-ink

Hyun-Jun Hwang, Kyung-Hwan Oh, and Hak-Sung Kim

1. In-situ measurement of temperature of Cu films

In this work, a polyimide (PI) film with 225 μm thickness was used as substrate material. In order to monitor the temperature of copper films in real time, a hole was drilled through the PI substrate using a pin; a thermocouple (type-K, 0.12 mm, Labfacility) was inserted into this hole and the prepared copper nanoparticle ink was printed on the PI substrate by covering the thermocouple using a doctor blade method (Fig. S1b). The sample size of coated copper nanoparticle film was determined as 2 cm x 2 cm; the thickness of the film was 40 μm.

To monitor temperature change during a flash light sintering process a few milliseconds in duration, a measurement apparatus was devised that combined a non-inverting amplifier circuit with an op-amp (LM 324N, STMicroelectronics), a power supply (SDP 30-3DT, SM Techno) and a type-K thermocouple with a response time of about 1 ms (Labfacility, UK) (Fig. S1a). The power supply was used to apply a constant voltage (15 V) to the non-inverting amplifier circuit to operate the op-amp. In the non-inverting amplifier circuit, the values of the resistances *R1* and *R3* were fixed at 10 kΩ, while *R2* and *R4* were fixed at 100 kΩ. During sintering, the output voltage (V_{out}) was recorded using an oscilloscope (DL1740E; Yokogawa) at 20 x 104 samples per second. Based on the non-inverting amplifier circuit, the temperature changes of the copper nanofilms during sintering can be calculated from the output voltage (V_{out}) by using the following equations:

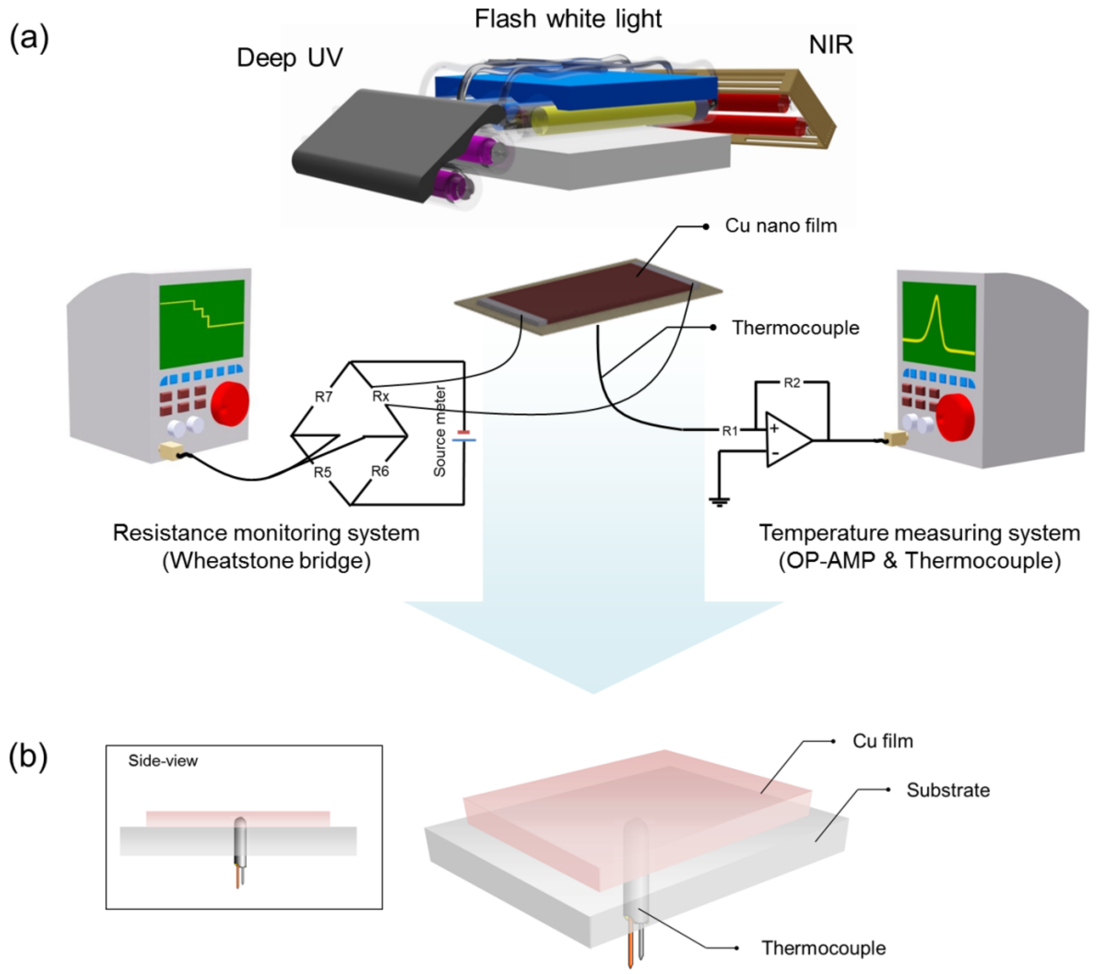

Fig. S1. The schematic diagrams of the flash light sintering, in-situ monitoring and in-situ temperature measuring system. (b) The schematics of Cu film specimen for temperature measurement in real time.

$$V_{out} = V_{in}\,[(1+R_2/R_1)(1+R_4/R_3)] \quad (1)$$

$$V_{in} = \alpha\,/\Delta T_{\,measured} \quad (2)$$

$$\Delta T_{\,measured} = V_{out}\,\alpha\,/[(1+R_2/R_1)(1+R_4/R_3)] \quad (3)$$

where V_{out} is the output voltage recorded by the oscilloscope, V_{in} is the voltage differential converted by the temperature gradient of the copper nanoparticle film, $\Delta T_{measured}$ is the temperature change of the copper nanoparticle film, and α is a correction factor, which was measured to be 0.025 ^{o}C/μV.

2. Transient heat transfer analysis

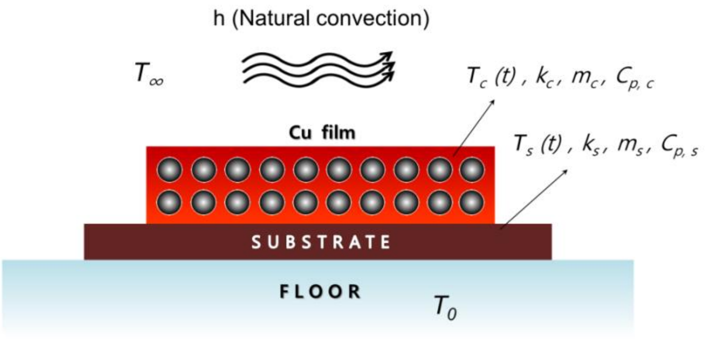

Fig. S2 The theoretical heat transfer model for calculation of the temperature of Cu nanofilms.

As shown in Fig. S2, the modeled heat transfer system was composed of the film, the PI substrate, and the floor. To calculate the temperature changes in this model, several assumptions were employed. First, radiation of heat from the film was neglected. Second, convection between the air surroundings and the film was assumed to take place through the mechanism of natural convection. Third, for simplicity, the values of the thermal properties of copper, PVP, and substrate were fixed at their specific values at room temperature, as listed in Table S1.

Hence, the general heat transfer equation can be written as follows:

$$q_{o,c} = h(T_c(t) - T_\infty) + k_c(\frac{T_c(t)-T_s(t)}{Lc}) + m_c C_{p,c}(\frac{dT_c(t)}{dt}) \quad (5)$$

$$q_{o,s} = k_s(\frac{T_s(t)-T_0}{L_s}) + k_c(\frac{T_s(t)-T_c(t)}{L_c}) + m_s C_{p,s}(\frac{dT_s(t)}{dt}), \quad (6)$$

where q_o is the heat flux, which is converted from the flash light power (the flash light energy measured using power meter, divided by the duration of flash light irradiation), T is the temperature, h is the natural convection coefficient, k is the thermal conductivity, L is the thickness, m is the mass, and C_p is the heat capacity. The subscripts c and s respectively denote the copper and the substrate.

Equations (5) and (6) can be rearranged to give $T_c(t)$ and $T_s(t)$:

$$\frac{dT_c(t)}{dt} = \frac{1}{\alpha}\,(cT_c(t) + dT_s(t) + e) \tag{7}$$

$$\frac{dT_s(t)}{dt} = \frac{1}{\beta}\,(dT_c(t) + fT_s(t) + g), \tag{8}$$

where $\alpha = m_c \cdot C_{p,c}$, $\beta = m_s \cdot C_{p,s}$, $c = -(k_c/L_c + h)$, $d = k_c/L_c$, $e = q_{o,c} + hT_0$, $f = k_c/L_c + k_s/L_s$ and $g = q_{o,s} + (k_s/L_s)T_0$. For the heat transfer calculations, the following initial conditions were assumed:

$$T_c(0) = T_\infty, \quad T_s(0) = T_0, \quad q_{o,s} = 0 \tag{9}$$

To solve the temperatures of the copper film and the PI substrate, the eigenvalues technique was employed, thereby obtaining the following homogeneous and particular solutions:

$$X_h = \begin{pmatrix} T_{h,c}\,(t) \\ T_{h,s}\,(t) \end{pmatrix} = C_1 \begin{pmatrix} d \\ -c + \lambda_1 \alpha \end{pmatrix} e^{\lambda_1 t} + C_2 \begin{pmatrix} d \\ -c + \lambda_2 \alpha \end{pmatrix} e^{\lambda_2 t} \tag{10}$$

$$X_p = \begin{pmatrix} T_{p,c}(t) \\ T_{p,s}(t) \end{pmatrix} = \frac{\alpha\beta}{cf - d^2} \begin{pmatrix} \frac{f}{\beta} & -\frac{d}{\alpha} \\ -\frac{d}{\beta} & \frac{c}{\alpha} \end{pmatrix} \begin{pmatrix} -\frac{e}{\alpha} \\ -\frac{g}{\beta} \end{pmatrix} , \tag{11}$$

where $T_{h,c}$ and $T_{h,s}$ are the homogeneous solutions for the temperatures of the copper film and the substrate, respectively; $T_{p,c}$ and $T_{p,s}$ are the particular solutions for the temperatures of the film and the substrate, respectively.

The temperature of the copper nanofilm and PI substrate was determined using the following combination of the homogeneous and particular solutions:

$$X = \begin{pmatrix} T_c(t) \\ T_s(t) \end{pmatrix} = X_h + X_p$$

$$= C_1 \begin{pmatrix} d \\ -c + \lambda_1 \alpha \end{pmatrix} e^{\lambda_1 t} + C_2 \begin{pmatrix} d \\ -c + \lambda_2 \alpha \end{pmatrix} e^{\lambda_2 t} + \frac{\alpha\beta}{cf - d^2} \begin{pmatrix} \frac{f}{\beta} & -\frac{d}{\alpha} \\ -\frac{d}{\beta} & \frac{c}{\alpha} \end{pmatrix} \begin{pmatrix} -\frac{e}{\alpha} \\ -\frac{g}{\beta} \end{pmatrix}, \qquad (12)$$

where $$\lambda_1 = [(\alpha f + \beta c) + \sqrt{(\alpha f - \beta c)^2 + 4\alpha\beta d^2}]/\, 2\alpha\beta \qquad (13\text{-a})$$

$$\lambda_2 = [(\alpha f + \beta c) - \sqrt{(\alpha f - \beta c)^2 + 4\alpha\beta d^2}]/2\alpha\beta \qquad (13\text{-b})$$

$$C_1 = \frac{1}{\alpha(\lambda_1 - \lambda_2)} [T_0 - \frac{1}{d}\left(T_\infty - \frac{dg - fe}{cf - d^2}\right)(-c + \lambda_2 \alpha) - \frac{de - cg}{cf - d^2}] \qquad (14\text{-a})$$

$$C_2 = \frac{1}{d}\left(T_\infty - \frac{dg - fe}{cf - d^2}\right) - \frac{1}{\alpha(\lambda_1 - \lambda_2)} [T_\infty - \frac{1}{d}\left(T_0 - \frac{dg - fe}{cf - d^2}\right)(-c + \lambda_2 \alpha) - \frac{de - cg}{cf - d^2}]. \qquad (14\text{-b})$$

In these heat transfer system equations, state changes of the copper nanoparticles (between solid and liquid) during the flash light irradiation are not considered. However, the copper nanoparticles could indeed melt and solidify due to flash light irradiation. Therefore, in the temperature calculation, we assumed further that if the calculated temperature of the copper nanoparticle film reached the melting temperature, the temperature of the film would remain constant until enough latent heat was gained to liquefy all of the copper nanoparticles simultaneously. The melting temperature of the copper nanoparticles was assumed to be about 215 ℃, because the average diameter of copper nanoparticle used in this work was about 40 nm [26]. In the calculation of the temperature increase during flash light irradiation, the energy for PVP vaporization was also considered in the latent heat analysis.

In the same manner, latent heat was considered when the nanoparticle film cooled after the flash light irradiation; the temperature of the film was assumed to stay at the melting temperature until enough latent heat was lost to solidify all the liquid copper nanoparticles simultaneously.

Table S1. Physical constants of copper, PVP and PI.

	Copper	PVP	PI
Specific gravity, ρ (kg/m^3)	8940	400	1430
Thermal conductivity, k (W/m· ^{o}C)	401	-	0.52
Heat capacity in the solid phase, $C_{p,s}$ (J/kg· ^{o}C) (in 1% DEG solution$^@$)	384.6	3360$^@$	1150
Heat capacity in the liquid phase, $C_{p,l}$ (J/kg· ^{o}C)	571.7	-	-
Bulk melting temperature T_m (^{o}C) (Transition temperature$^{\#}$)	1084.6	160$^{\#}$	420
Latent heat of fusion, L (J/kg)	2.05 ×10^5	-	-

*Natural convection (h) : 30 W/m^2·K

3. The experimental results and theoretical results of Cu films during the photonic sintering process

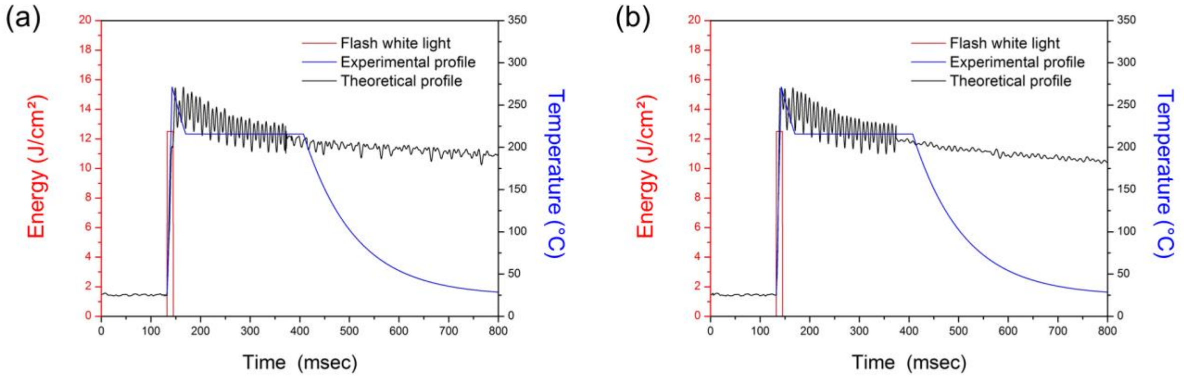

Fig. S3 The experimental results of in-situ measured temperature of copper films and theoretical results of heat transfer calculation; (a) the flash white light sintering (12.5 J/cm^2 irradiation energy, 1 pulse of 10 ms pulse duration) and (b) the deep UV-assisted flash white light sintering (deep UV: 30 mW/cm^2).

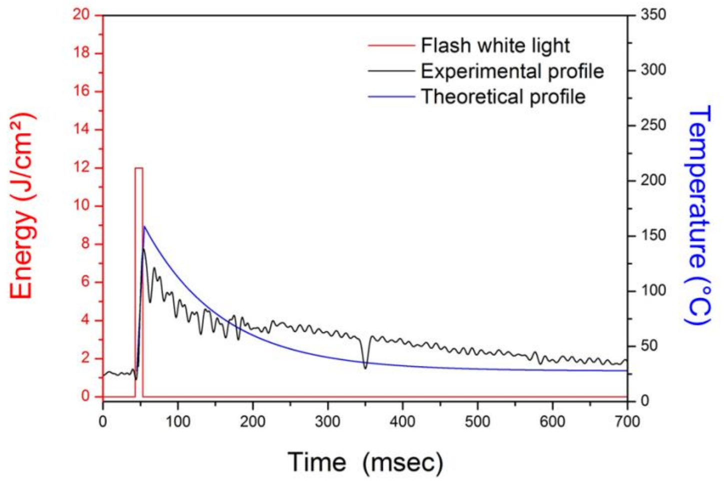

Fig. S4 The experimental and theoretical temperature profiles of PI substrate during the deep UV-assisted flash white light sintering process (flash white light: 12.5 J/cm^2 irradiation energy, 1 pulse of 10 ms pulse duration, 30 mW/cm^2 of deep UV power).

4. The cross-sectional profile of the sintered Cu pattern

The thickness of Cu films were measured by Alpha step (KLA Tencor AS500) to calculate resistivity. The cross-sectional profile of the sintered Cu pattern was shown in Fig.S5. It was observed that the flash light sintered Cu pattern has thickness of 40 μm.

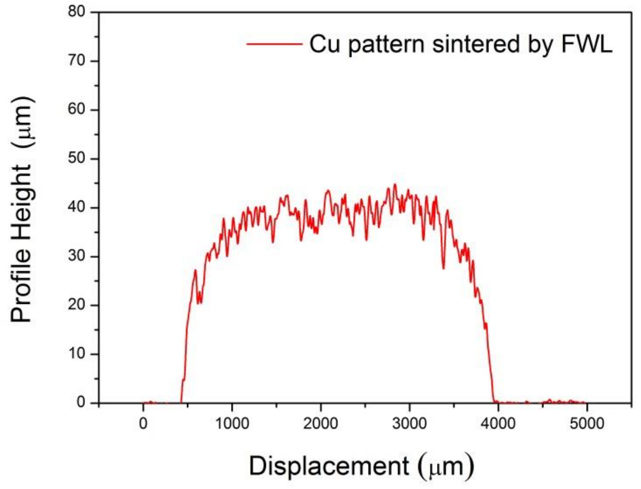

Fig. S5 The line cross-sectional profiles of Cu film after flash white light sintering.
